# Seroprevalence of hepatitis C virus antigen in patients with chronic liver disease and hepatocellular carcinoma at 12^th^ week of treatment: a cross-sectional study

**DOI:** 10.11604/pamj.2022.43.72.35450

**Published:** 2022-10-11

**Authors:** Donatien Serge Mbaga, Jacky Njiki Bikoï, Etienne Atenguena Okobalemba, Justin Olivier Essindi, Chris André Mbongue Mikangue, Alexandra Emmanuelle Membangbi, Aïcha Ngoutane, Arnaud Franck Elang, Sabine Aimée Touangnou-Chamda, Carole Stéphanie Sake, George Ikomey Mondinde, Sebastien Kenmoe, Sara Honorine Riwom Essama

**Affiliations:** 1 Department of Microbiology, The University of Yaoundé I, Yaoundé, Cameroon,; 2 Faculty of Medicine and Biomedical Sciences, The University of Yaoundé I, Yaoundé, Cameroon,; 3 Institute of Medical Research and Medicinal Plant Study, Yaoundé, Cameroon,; 4 Faculty of Medicine and Biomedical Sciences, The University of Yaoundé I (Center for the Study and Control of Communicable Diseases (CSCCD), Yaoundé, Cameroon,; 5 Department of Microbiology and Parasitology, University of Buea, Buea, Cameroon

**Keywords:** Hepatitis C virus Ag, chronic hepatitis, cirrhosis, hepatocellular carcinoma

## Abstract

**Introduction:**

epidemiological data suggests that more than 50% of hepatitis C virus (HCV) patients fail treatment. The objective of the study was to highlight the seroprevalence of hepatitis C virus antigen (HCV Ag) at the 12^th^ week of treatment.

**Methods:**

during a cross-sectional study, participants with chronic liver disease and hepatocellular carcinoma (HCC) were recruited between December 2020 and March 2022 at the Yaoundé General Hospital (HGY) and the University Teaching Hospital of Yaounde (UTHY). Five millilitres of blood samples were taken from each consenting participant and then a qualitative search for HCV Ag by Enzyme-Linked Immuno Assay (ELISA) was performed. Analysis of the results was performed using SPSS Version 25.0 software.

**Results:**

out of the 192 participants selected for the study, only 92 (47,9%) participants were at 12 weeks of treatment, including 69 (75%) participants positive for the hepatitis C virus antibody (HCV Ab) by RDT. Of these participants, 44 (47.8%) participants were positive for HCV Ag by ELISA, respectively 19/37 (51.3%), 14/19 (73.6%), 11/13 (84.6%) with chronic hepatitis (HC), Cirrhosis, and HCC (P<0.0001).

**Conclusion:**

our results showed a high prevalence of HCV Ag in patients at their 12^th^ week of treatment which predicts treatment failure and calls for public policy to develop new management strategies to prevent HCV treatment failure in our context.

## Introduction

Hepatitis C virus (HCV) is one of the main viruses responsible for liver disease [1]. An estimated 71.1 million people are living with HCV infection worldwide [2]. Chronic hepatitis C infection is the leading cause of death in people with chronic hepatitis (CH), cirrhosis, and hepatocellular carcinoma (HCC) [3], and this chronic infection causes 25% of HCC cases [4]. WHO Africa estimates that HCV is one of the viruses responsible for 96% of mortality in people with cirrhosis and those with HCC [5]. In Cameroon, HCV affects 6.5% of people [6] and shifts considerably across the population in the country according to the most recent Cameroonian studies [7-13]. The primary objective of HCV treatment with direct-acting antivirals (DAAs) is to cure infected people. This healing can be measured by the sustained virological response (RSV), which is defined as an absence of detectable HCV ribonucleic acid (RNA) in the serum at the 12^th^ or 24^th^ week of treatment [14]. Current DAA-based treatments have shown promising results, with a cure rate of more than 95% of patients from the 8^th^ week of treatment [15]. This prompted the World Health Organization (WHO) to establish a global strategy for the elimination of viral hepatitis which aims to reduce the number of new infections, and deaths and increase by 80% the proportion of people treated by 2030 [3]. Unfortunately, some studies have shown that more than 50% of HCV patients fail treatment and systematically relapse and later develop treatment resistance [16,17]. This is mainly observed in certain hard-to-cure populations, such as patients with severe hepatic decompensation, active HCC, chronic HCV genotype 3 infection, and those multiple DAA failures [18].

To date, multiple obstacles that may hinder the success of HCV treatment have been identified [19] and constitute the major limiting factors that may contribute to a significant increase in HCV morbidity and mortality over the next 15 years in developing countries if nothing is done [20]. Screening and diagnosis of HCV infection rely on three different approaches which can be used alone or in combination. These are the detection of an HCV IgG antibody (HCV-Ab), the detection of the capsid antigen of the HCV (HCV-Ag) which is highly retained from one viral strain to another, and the detection of HCV RNA. Of these approaches only HCV Ag and RNA can confirm an active infection [21] even the recent WHO guidelines continue to recommend the use of the Ac-HCV test for first-line screening [22]. Hepatitis C virus Ag in serum or plasma is an indirect marker of HCV replication that can be used as an alternative to HCV RNA to diagnose HCV viremia at the 12^th^ or 24^th^ week of treatment to assess whether the patient eliminates HCV in a context where HCV viral load assessment is very expensive for poor people [23]. We therefore set the objective to highlight the seroprevalence of HCV Ag in patients with chronic liver disease and hepatocellular carcinoma at the 12^th^ week of treatment.

## Methods

**Study design and setting:** this study was cross-sectional. It took place in the city of Yaoundé, and the hepato-gastroenterology departments of the Yaoundé General Hospital (YGH) and internal medicine of the University Teaching Hospital of Yaoundé (UTHY) served as a recruitment site for participants between December 2020 and March 2022.

**Study population and sampling:** all participants were informed of the aim of the study before their inclusion in the study. The study population was composed of participants with chronic liver disease and those with hepatocellular carcinoma (HCC) who came for follow-up at the 12^th^ week of treatment either the UTHY or the HGY, the consenting participants were divided into 3 groups. Group 1 consisted of participants with chronic hepatitis C (CHC) who were negative for cirrhosis or HCC. Group 2 consisted of participants with cirrhosis diagnosed by radiographic, biochemical, and clinical examinations and confirmed by a physician. Group 3 was composed of participants with HCC diagnosed by histological, radiographic, and biochemical examinations and confirmed by a physician. These participants were chosen, regardless of their sex, age (21 years or older), nationality, and treatment. Any participant who was having others aetiologies or comorbidities was excluded from the study.

**Procedure for collecting socio-demographic and clinical data:** data collection from each participant was done consecutively and prospectively. Each of the participants spoke in the consultation box with the principal investigator. The information leaflet was presented, to those unable to read, the principal investigator read the contents of the leaflet and then explained the aim of the study. The consenting patient gave his signed agreement to participate in the study. Then a pre-established and standardized questionnaire was administered to him.

**Variables collected:** for each participant, qualitative and quantitative variables were collected either by interview or blood samples analysis. The qualitative variables collected were: sex, marital status, scarification, tattoos, piercing, alcohol consumption before disease; tobacco consumption before disease; surgical history; history of blood transfusion; the treatment submitted, patients with chronic hepatitis; cirrhosis; HCC; HCV-Ab; HCV-Ag. The quantitative variables collected were age; alanine aminotransferase (ALT) glutamic-pyruvic transaminase (GPT); aspartate aminotransferase (AST) glutamic-oxaloacetic transaminase (GOT); alpha-fetoprotein (AFP).

**Collection and fate of blood samples:** overall, five millilitres of blood samples were taken from the crook of the elbow of each consenting participant, in ethylenediamine tetraacetic acid (EDTA) tubes. The samples were transferred to the Centre for the Study and Control of Communicable Diseases (CSCCD) of the Faculty of Medicine and Biomedical Sciences of Yaoundé (FMBS) where they were divided into three aliquots of the same volume each. The first aliquot was used for the search for HCV Ab and HCV Ag, the second aliquot was used for the quantification of ALT, AST, and AFP. The third aliquot was divided into 2 parts and then saved for further analysis.

### Biological analysis of samples

**Search for hepatitis C virus antibody and hepatitis C virus antigen:** we carried out a qualitative search for HCV-Ab by immunochromatography according to the designers of rapid diagnostic tests (RDT) VOXTUR BIO LTD for HCV manufactured in India. Only positive results were confirmed by Monolisa™ HCV Ag-Ab ultra for the detection of capsid antigen of HCV in human plasma manufactured in the USA. Samples whose optical density was greater than or equal to the threshold (ratio ≥1) were considered positive. We did not retest samples due to a lack of reagent.

**Alpha-fetoprotein, alanine aminotransferase, and aspartate aminotransferase quantification:** quantification of AFP and transaminases (ALT and AST) was carried out only on ELISA-positive samples. Indeed, 30 HCV were selected randomly using a random number generator in Microsoft Excel. The codes corresponding to the aliquots were used for analyses. alpha-fetoprotein quantification was performed using Finecare™ Rapid AFP quantitative Strip (made in China) which uses a fluorescence immunochromatography Assay. Quantification of transaminases (ALT and AST) was performed using sprinreact reagents (made in Spain).

**Ethical considerations:** to respect the ethics of medical research, the study was authorized by the University Teaching Hospital of Yaoundé (N° 3235/AR/CHUY/DG/DGA/CAPRC), Yaoundé General Hospital (N/Ref: 213-21/HGY/DG/DPM/MA-TR). The CSCCD allowed the laboratories analysis. Each patient who participated in this study read the information sheet then gave their signed consent and finally an anonymity code was assigned to them.

**Sample size calculation:** the minimum sample size was 148 participants. The calculation of this sample size was made using the prevalence of HCV, which are 6.5% in Cameroon [6], and according to GLOBOCAN data from 2020 which provided a prevalence of 4.2% for liver cancer in Cameroon [24]. We used the following formula [25]:


$$n=\frac{P(1-P){\left(Z_{1-\alpha }\right)}^{2}}{i^{2}}$$

Z = the level of statistical significance with a 95% confidence interval (CI) of 1.96; i= the level of precision of 0.05; P =prevalence of outcomes.

**Statistical analysis:** for each participant recruited, qualitative and quantitative variables were collected by either interviewing the participant or analyzing blood samples. The collected data were recorded and processed using Excel Version 2016 (Microsoft Corp., USA). Analyses were done using the biostatistical software Statistical Package for Social Sciences (SPSS) Version 25.0. The Chi-square test allowed us to compare the proportions between the different groups and the non-parametric Kruskal Wallis test allowed us to compare the means ± standard deviations between the different groups. A p-value of < 0.05 was considered statistically significant.

## Results

**Figure 1:**
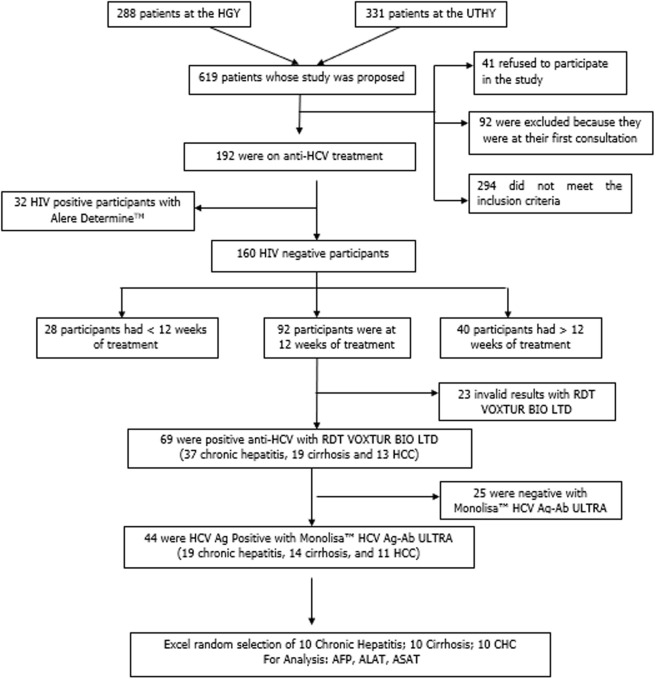
flow Chart of selection of participants

**Participants:** we offered the study to 619 patients who came for consultation at the UTHY or the YGH, respectively 331 patients at the UTHY and 288 patients at the YGH. Of these 619 patients, 41 refused to participate in the study, which gave a non-response rate of 6.6%, 92 were excluded because they were on their first day of consultation and 294 did not meet the inclusion criteria. The remaining 192 participants were on anti-HCV treatment, of which 32 were positive for Human Immunodeficiency Virus (HIV) and were excluded. Of the remaining 160 participants, 28 were at less than twelve weeks of treatment, 92 were at 12 weeks of treatment and 40 were at more than 12 weeks of treatment. Among the patients who were at 12 weeks of treatment, 69 (75%) tested positive for HCV Ab, out of these participants, only 44 (47.8%) were positive for HCV Ag (19 CH, 14 cirrhosis, and 11 HCC). We randomly selected 10 chronic hepatitis, 10 cirrhosis, and 10 HCC samples to quantify AFP and transaminases (Figure 1).

**Sociodemographic and clinical characteristics of study participants:** overall, participants with chronic hepatitis (CH) were the majority with 19 HCV-positive participants. But the HCC group had the highest average age with 65.8 ± 4.2 years. The 50-59 and 60-69 age groups were the most represented with 8 (42.1%) and 8 (72.7%) respectively for CH and HCC. The male gender 11 (57.9%) of the CH group was most represented. According to marital status, married participants were the most represented in the CH group with 12 (63.1%). For scarification, the CHC group was the most represented with 4 (36.3%) and 5 (26.3%) of CH participants having tattoos. Seven (36.8%) CH participants reported that they consumed alcohol before their illness while only 2 (18.1%) HCC participants reported smoking cigarettes before their illness. Two participants both CH and Cirrhosis groups respectively (10.5) and (14.2%) had received a blood transfusion before their disease. Concerning anti-HCV treatment, 8 (42.1%) participants were taken sofosbuvir + ribavirin (Table 1).

**Table 1:** sociodemographic and clinical characteristics of HCV Ag positive participants after 12 weeks of treatment

	n = 44 HCV		
	HC n =19	Cirrhosis n =14	HCC n =11
	n ( %)	n ( %)	n ( %)
**Age**			
Mean±standard deviation	49.7±7.3	56.5±8.2	65.8±4.2
20-29	//	//	//
30-39	2 (10.5)	//	//
40-49	7 (36.8)	4 (28.5)	//
50-59	8 (42.1)	5 (35.7)	//
60-69	2 (10.5)	4 (28.5)	8 (72.7)
70-79	//	1 (7.1)	3 (27.2)
**Gender** (male)	11 (57.9)	8 (57.1)	10 (90.9)
**Marital status**			
Married	12 (63.1)	10 (71.4)	8 (72.7)
Single	5 (26.3)	4 (28.5)	//
Widower	2 (10.5)	//	//
Scarification (yes)	2 (10.5)	3 (21.4)	4 (36.3)
Tattoo (yes)	5 (26.3)	3 (21.4)	//
Alcohol consumption before illness (yes)	7 (36.8)	3 (21.4)	3 (27.2)
Smoking before illness (yes)	1 (5.2)	1 (7.1)	2 (18.1)
Blood transfusion (yes)	2 (10.5)	2 (14.2)	1 (9.0)
**HCV treatment**			
sofosbuvir + ribavirin	8 (42.1)	5 (35.7)	4 (36.3)
sofosbuvir + ribavirin + pegylated interferon alpha	7 (36.8)	5 (35.7)	2 (18.1)
sofosbuvir + ledispavir	4 (21.0)	4 (28.5)	5 (45.4)

HCC: hepatocellular carcinoma; CH: chronic hepatitis; HCV: hepatitis C virus

**HCV profile of study participants:** out of the 192 participants on HCV antiviral treatment, 92 were HIV-negative at the 12^th^ week of their treatment. Of these participants, 58 had chronic hepatitis (CH), 21 had cirrhosis, and 13 had HCC. Sixty-nine participants were positive for the HCV Ab test respectively 37/58 (63.7%) CH; 19/21 (90.4%) Cirrhosis, and 13/13 (100%) HCC (P<0.009), and 23 tests were invalid results. We excluded these samples from our analysis. Out of positive HCV Ab participants, only 44 were positive for HCV Ag with ELISA with optical densities equal to or greater than the threshold, ratios ≥ 1 (Table 2). These samples were not retested following a lack of reagents. They were declared positive for HCV in this study respectively 19/58 (51.3%) CH, 14/21 (73.6%) Cirrhosis, and 11/13 (84.6%) HCC (P<0.000 1) (Table 3).

**Table 2:** profile of HCV markers sought in participants at 12 weeks of treatment

	Profile of HCV Ab markers sought with RDTs on 92 participants at 12 weeks of treatment				HCV Ag markers were searched with ELISA only on the 69 RDT-positive participants			
	HC n =58 (%)	Cirrhosis n =21(%)	HCC n =13 (%)	P-value	HC n =37 (%)	Cirrhosis n =19 (%)	HCC n =13 (%)	P-value
Undetermined	21	2	//		//	//	//	
Negative test	//	//	//	0.009	18	5	2	<0.000 1
Positive test	37 (63.7)	19 (90.4)	13 (100)		19 (51.3)	14 (73.6)	(84.6)	

With ELISA, samples with a ratio <1 were considered negative; Samples with a ratio ≥ 1 were considered positive; HCC: hepatocellular carcinoma; CH: chronic hepatitis; HCV: hepatitis C virus

**Table 3:** seroprevalence of HCV Ag tested in participants at 12 weeks of treatment

Attendees	n	HCV Ag/Anti-HCV n (%)
Chronic hepatitis	58	19/58 (32.7%)
Cirrhosis	21	14/21 (66.6%)
Hepatocellular carcinoma	13	11/13 (84.6%)

**Biochemical markers of HCV-positive participants simultaneously on RDT and ELISA:** participants whose samples were ELISA positive were randomly selected. Each group was made up of 10 participants. Transaminase was tested for each group ALT (GPT) and ASAT (GOT) were higher in participants with HCC (75.4±7.3 IU/L and 72.2 ± 13.8 IU/L respectively) compared to participants with CH and Cirrhosis, with P<0.0001. Regarding AFP, participants with HCC had also a higher mean of AFP of 249.8±6.1 ng /ml, followed by participants with cirrhosis 82.3±10.7 ng /ml, and finally participants with CH 58.5±26.3 ng /ml (P<0.0001) (Table 4).

**Table 4:** biochemical markers of HCV Ag positive participants by ELISA at 12 weeks of treatment

	Chronic hepatitis (n=10)	Cirrhosis (n=10)	Hepatocellular Carcinoma (n=10)	p-value
ALT (GPT) IU/L	29.0±9.8	58.7±5.1	75.4±7.3	<0.000 1
AST (GOT) IU/L	23.1±6.1	53.7±8.6	72.2±13.8	<0.000 1
AFP ng /ml	58.5±26.3	82.3±10.7	249.8±6.1	<0.000 1

alanine aminotransferase (ALT) glutamic-pyruvic transaminase (GPT); aspartate aminotransferase (AST) glutamic-oxaloacetic transaminase (GOT); alpha-fetoprotein (AFP); hepatitis C virus antigen (HCV Ag)

## Discussion

By 2030, with the treatment of hepatitis C using DAAs, the WHO goal is to eliminate HCV. Therefore, unsatisfactory HCV treatment outcomes must be reported to facilitate better management of those seeking care. This study aimed to estimate the seroprevalence of HCV Ag in participants with liver disease and HCC at the 12^th^ week of treatment. Of the 69 samples positive for HCV Ab with the RDT, only 44 samples were positive for HCV Ag with ELISA, respectively 19/37 (51.3%) HC, 14/19 (73.6%) Cirrhosis, 11/13 (84.6%) HCC (P<0.0001). These results show a high prevalence of HCV Ag, which translates into a long and very expensive treatment for patients. Since some patients achieve healing rather at the 24^th^ week this would mean that the treatment of these patients will have to be prolonged until the 24^th^ week. Since treatment against HCV is expensive in our context, this appeals to public authorities to bear the costs of treatment more because infected patients come from the most disadvantaged sections of the population. In other words, to remain in the policy of eradicating HCV, the governments of developing countries must set up solid policies for managing the treatment of people with HCV so that they can achieve the sustained virologic response (SVR). This implies that patients whose HCV Ag results by ELISA are positive at the 12^th^ week of their therapy require special follow-up. Indeed, several studies have been interested in assessing the role of HCVA in the diagnosis of hepatitis C. It has been demonstrated that the HCV Ag marker can be used to diagnose hepatitis C and of evaluating HCV SVR [3] because there is a correlation between the titter of HCVAg and the viral load of HCV, which makes the capsid antigen a marker of viremia and viral replication of HCV [23]. It, therefore, remains important to carefully follow up with people receiving HCV treatment so that increased efforts can be made to identify people who are successful or unsuccessful to converge toward the international HCV elimination goals. Although HCV-Ag testing can potentially replace viral load for HCV diagnosis or monitoring in some settings, national and international guidelines still advocate the use of PCR as the gold standard test for monitoring progress during and after treatment and is currently still recommended [21].

Our results may have been underestimated given the fact that we only considered ELISA positive for all samples whose optical density was greater than or equal to the threshold (ratio ≥ 1) and that we did not retest the samples with the threshold (ratio between 0.9 and 1). Indeed, it is known that the treatment of hepatitis C using DAAs very rarely eliminates HCV in all people treated. This is probably due to certain risk factors such as on the one hand the advanced age and health status of the patient and on the other hand, the cost, the duration, and the missed doses can negatively affect the results of the treatment [23,26-28]. Added to this, the lack of inexpensive viral diagnostic tests and universally accessible treatment adapted to resource-limited settings represent an obstacle to the implementation of HCV surveillance in difficult contexts [22].

Regarding biochemical markers to assess the liver health status of study participants at their 12^th^ week of treatment, CHC participants had the highest ALT (GPT), AST (GOT), and AFP. high with respectively 75.4±7.3 IU/L; 72.2±13.8 IU/L; 249.8±6.1 ng/mL compared to participants with CH and Cirrhosis (P<0.0001). The transaminase test (ALT and AST) is simple, practical, inexpensive, and non-invasive but not specific to liver disease. Indeed, transaminases are present respectively in the cytoplasm and mitochondria of hepatocytes and muscle cells. When hepatocytes are damaged by the cytopathic effects of oncogenic hepatitis viruses, transaminases are released into the bloodstream resulting in increased serum levels of ALT and AST in the peripheral blood. It is also possible that inflammatory stimulation due to tumour progression stimulates hepatocytes to produce abundant amounts of transaminases compared to what would be found in patients with early liver disease [29]. Besides its role in the diagnosis of HCC, higher serum AFP levels were also observed in participants with HCC. Alpha-fetoprotein is currently the most widely used serum biomarker for screening and early diagnosis of HCC, as well as for evaluating the efficacy and prognosis of HCC treatment. However, not all HCC tumours contribute to an increase in AFP. This makes this marker non-specific to the CHC facility. It is therefore important to explore the search for new biomarkers capable of overcoming the shortcomings of transaminases and AFP to complement X-ray examinations in the hope of achieving HCC screening rates close to those that would be obtained using the biopsy [22,30].

The results obtained in our study can be used to update and add epidemiological data to influence policymakers in the policy of prevention and control of HCV during treatment in Cameroon. We excluded all patients with comorbidities such as diabetes, and HIV because these could play a major role in the ineffectiveness of the treatment. Only plasma samples were considered in this work, we could have obtained more revealing results if we had used the serum, whole blood, or biopsy fragments. The fact of not having retested positive samples as indicated by the Monolisa™ HCV Ag-Ab ultra-protocol constitutes a limitation insofar as certain samples declared positive would have been negative and vice versa. As other limitations, we can note the lack of staging of cirrhotic participants and those with HCC. To achieve the WHO objectives plan to eliminate HCV by 2030, sub-Saharan Africa, in particular Cameroon, will have to actively prioritize the implementation of several strategies such as the reduction or even total coverage of treatment costs. of HCV by public authorities. Identify groups at high risk of therapeutic failure, so that they benefit from special attention in the management of their treatment and indirectly prevent possible resistance to antiviral treatment, and monitor the rebound of viral load and the diversion towards occult hepatitis C.

## Conclusion

Our results showed a high prevalence of HCV Ag in patients with liver disease and hepatocellular carcinoma at their 12^th^ week of treatment, suggesting a high prevalence of early treatment failure. These results challenge public policies to develop treatment management strategies to prevent treatment failure and treatment desertion due to Anti HCV treatment costs in our context and encourage the development of the vaccine against HCV. All this is to prevent mortality from cirrhosis, liver failure, and hepatocellular carcinoma.

### What is known about this topic

- Sustained virological response measured at 12^th^ or 24^th^ weeks of treatment;
- More than 50% of HCV patients fail treatment.

### What this study adds

- Highlight a high prevalence of HCV Ag at 12 weeks of treatment.
